# The usefulness of systemic inflammatory markers as diagnostic indicators of the pathogenesis of diabetic macular edema

**DOI:** 10.5935/0004-2749.20200051

**Published:** 2020

**Authors:** Cagri Ilhan, Mehmet Citirik, Mehmet Murat Uzel, Hasan Kiziltoprak, Kemal Tekin

**Affiliations:** 1 Department of Ophthalmology, Hatay State Hospital, Hatay, Turkey; 2 Department of Ophthalmology, University of Health Sciences, Ankara Ulucanlar Eye Education and Research Hospital, Ankara, Turkey; 3 Department of Ophthalmology, Balikesir University, Balikesir, Turkey; 4 Department of Ophthalmology, Bingol Maternity and Child Hospital, Bingol, Turkey; 5 Department of Ophthalmology, Ercis State Hospital, Van, Turkey

**Keywords:** Macular edema, Diabetic retinopathy, Mean platelet volume, Lymphocyte count, Neutrophils, Inflammation, Edema macular, Retinopatia diabética, Volume plaquetário médio, Contagem de linfócitos, Neutrófilos, Inflamação

## Abstract

**Purpose:**

To investigate the usefulness of systemic inflammatory markers [i.e., white blood cell and platelet counts, mean platelet volume, and their ratios] as diagnostic markers of the pathogenesis of diabetic macular edema.

**Methods:**

The study cohort included 80 diabetic macular edema patients (40 with diabetic retinopathy and 40 without) and 40 healthy ageand sex-matched controls. Neutrophil, lymphocyte, monocyte, and platelet counts, and the mean platelet volume were determined from peripheral blood samples, and the monocyte/lymphocyte, platelet/lymphocyte, and mean platelet volume/lymphocyte, and neutrophil/lymphocyte ratios were calculated and compared among groups.

**Results:**

The mean neutrophil/lymphocyte ratio of the diabetic macular edema and non-diabetic macular edema groups was higher than that of the control group, and the value of the diabetic macular edema group was higher than that of the non-diabetic macular edema group (p<0.001 in diabetic macular edema vs. control, p=0.04 in non-diabetic macular edema vs. control, and p=0.03 in diabetic macular edema vs. non-diabetic macular edema). A neutrophil/lymphocyte cutoff value of ≥2.26 was identified as an indicator of the pathogenesis of diabetic macular edema with a sensitivity of 85% and specificity of 74%. The mean platelet volume of the diabetic macular edema group was higher than those of the non-diabetic macular edema and control groups, while those of the non-diabetic macular edema and control groups were similar (diabetic macular edema vs. non-diabetic macular edema, p=0.08; diabetic macular edema vs. control, p=0.02; and nondiabetic macular edema vs. control, p=0.78). All other parameters were similar between groups (all p>0.05).

**Conclusion:**

The neutrophil/lymphocyte ratio and mean platelet volume of the diabetic macular edema group were higher than those of the non-diabetic macular edema and control groups. A neutrophil/lymphocyte ratio cutoff value of ≥2.26 was identified as an indicator of the pathogenesis of diabetic macular edema with high sensitivity and specificity. Moreover, the neutrophil/ lymphocyte ratio of the non-diabetic macular edema group was higher than that of the control group.

## Abstract (pt)

**Objetivo:**

Investigar a utilidade de marcadores inflamatórios sistêmicos (ou seja, contagem de glóbulos brancos e plaquetas, volume médio de plaquetas e suas proporções) como marcadores de diagnóstico da patogênese do edema macular diabético.

**Métodos:**

A coorte do estudo incluiu 80 pacientes com edema macular diabético (40 com retinopatia diabética e 40 sem) e 40 controles saudáveis de acordo com a idade e sexo. As contagens de neutrófilos, linfócitos, monócitos, plaquetas e valores do volume plaquetário médio foram determinados a partir de amostras de sangue periféricdo, e as proporções de monócitos/linfócitos, plaquetas/linfócitos, volume plaquetário médio/linfócitos e neutrófilos/linfócitos foram calculadas e comparadas entre os grupos.

**Resultados:**

A proporção média de neutrófilos/linfócitos dos grupos com edema macular diabético e não-diabético foi maior que a do grupo controle, e o valor do grupo com edema macular diabético foi maior que o do grupo com edema macular não diabético (p<0,001 no com edema macular diabético vs. controle, p=0,04 no com edema macular não diabético vs. controle e p=0,03 no com edema macular dia bético vs. o com edema macular não-diabético). Um valor de corte de neutrófilos/linfócitos ≥2,26 foi identificado como um indicador da patogênese do edema macular diabético com sensibilidade de 85% e especificidade de 74%. O volume plaquetário médio do grupo com edema macular diabético foi maior que o dos grupos com edema macular não-diabético e controle, enquanto os do grupo de edema macular não-diabético e controle foram semelhantes (edema macilar diabético vs. Edema macular não-diabético, p=0,08; com edema macular diabético vs. controle, p=0,02; e com edema macular não-diabético vs. controle, p=0,78). Todos os outros parâmetros foram semelhantes entre os grupos (todos p>0,05).

**Conclusão:**

A proporção de neutrófilos/linfócitos e o volume plaquetário médio do grupo com edema macular diabético foram superiores aos do grupo com edema macular não-diabético e controle. Um valor de corte da razão neutrófilos/linfócitos ≥2,26 foi identificado como um indicador da patogênese do edema macular diabético com alta sensibilidade e especificidade. Além disso, a proporção de neutrófilos/linfócitos do grupo com edema macular não-diabético foi superior à do grupo controle.

## INTRODUCTION

Diabetic macular edema (DME) is an important cause of visual loss at any stage of diabetic retinopathy (DR)^(1,2)^. Disruption of the blood-retinal barrier due to capillary dilation, micro-aneurysm formation, and pericyte loss results in fluid leakage into the retinal layers and subsequent macular thickening^(3)^. The angiogenic, inflammatory, and oxidative stress pathways play important roles in the pathophysiology of DME. According to the inflammatory hypothesis, leukocyte adhesion to the vascular endothelium (leukocytes adhere more tightly in a hyperglycemic environment) directly increases vascular permeability and damages endothelial cells through the release of free radicals, enzymes, and cytokines^(4-6)^. Platelets and erythrocytes also contribute to this process through capillary occlusion produced by cellular thrombi^(7)^. Retinal ischemia and hypoxia stimulate further migration of inflammatory cells, the formation of reactive oxygen species, and the release of angiogenic growth factors^(8)^.

White blood cell (WBC) (including neutrophils, lymphocytes, and monocytes) and platelet counts, the mean platelet volume (MPV), and their ratios are useful indicators of systemic low-grade inflammation^(9)^. The superiority of the neutrophil/lymphocyte ratio (NLR) to total leukocyte count has been demonstrated in previous studies^(10,11)^. Although the roles of NLR in various systemic diseases have been widely reported, possible relationships with ocular diseases associated with ocular or systemic inflammation remain unclear^(12-15)^.

Therefore, the aim of the present study was to investigate the usefulness of systemic inflammatory markers (SIMs), including WBC and platelet counts, MPV, and their ratios, as diagnostic indicators of the pathogenesis of DME.

## METHODS

This prospective study was conducted in the Retina Unit of the Ophthalmology Department of a single tertiary hospital between July 2017 and November 2017. The study protocol was approved by the local ethics committee and conducted in accordance with the tenets of the Declaration of Helsinki. All patients received a verbal explanation of the nature of the study prior to providing written informed consent.

### Study subjects

The study cohort included 80 type 2 diabetic patients with non-proliferative DR: 40 with DME (DME group) and 40 without DME (non-DME group). All patients had high glycated hemoglobin levels (6.5%-8.5%) and all were receiving insulin therapy. The absence of acute inflammation, infection, renal insufficiency, connective tissue diseases, and inflammatory bowel diseases was confirmed by an experienced internist. Detailed ophthalmological examinations, which included visual acuity, Goldmann applanation tonometry, slit-lamp biomicroscopy, and fundoscopy after pupillary dilatation, were performed for all patients. The status of retinopathy and macular edema was assessed by fundus photography, fluorescein angiography, and optical coherence tomography. In accordance with The Early Treatment of Diabetic Retinopathy Study (ETDRS)^(16)^ and the International Clinical Diabetic Retinopathy Disease Severity Scale^(17)^, the criteria for inclusion into the DME and non-DME groups included any severe or moderate non-proliferative DR condition, as follows: 1) >20 intraretinal hemorrhages in each of four quadrants, 2) definite venous beading in two or more quadrants, 3) prominent intraretinal microvascular abnormality in one or more quadrants, and 4) more than just microaneurysms, but less than the severe non-proliferative DR criteria in this list. The subjects in the DME group additionally had DME, as demonstrated by the presence of increased central retinal thickness due to cystoid changes in horizontal cross-sections of the central fovea, and as confirmed by optical coherence tomography, as well as any of the following Early Treatment of Diabetic Retinopathy Study (ETDRS)^(18)^ criteria: 1) retinal thickening at or within 500 mm from the center of the macula, 2) hard exudates at or within 500 mm from the center of the macula if accompanied by thickening of the adjacent retina, and 3) a zone of retinal thickening, one disc area of larger in size, located one disc diameter or less from the center of the macula. Patients who underwent vitreoretinal surgery or intravitreal injection, or with other active or past ocular conditions, including severe dry eye, keratoconus, iridocyclitis, glaucoma, retinal vascular diseases except DR, signs of proliferative retinopathy, age-related macular degeneration, central serous chorioretinopathy, and optic neuropathies, were excluded from the study. Diabetics with systemic and/or ocular co-morbidities with simulating ophthalmic manifestations and proliferative retinopathies associated with other systemic diseases were also excluded. As a control group, 40 ageand sex-matched healthy subjects were included after similar detailed ophthalmological and systemic evaluations.

### Evaluation of blood cell parameters

Neutrophil, lymphocyte, monocyte, and platelet counts, and MPV were obtained peripheral blood samples using an ABX Pentra DX 120 Hematology Analyzer (Horiba, Inc., Kyoto, Japan). The NLR and monocyte/lymphocyte, platelet/lymphocyte, and MPV/lymphocyte ratios were calculated by dividing the count of neutrophils, monocytes, platelets, and MPV by the count of lymphocytes, respectively.

### Statistical analysis

All statistical analyses were conducted using IBM SPSS Statistics for Windows, version 22.0. (IBM Corporation, Armonk, NY, USA). The mean age and female/male ratio of the groups are presented as descriptive data. The mean WBC counts and calculations of defined variables are presented in table form. Differences among the three groups were evaluated using one-way analysis of variance and a probability (p) value of ≤0.017 was considered statistically significant, after Bonferroni correction (0.05/3 = 0.017). The Mann-Whitney U test was used for post-hoc analysis of two independent samples, for which a p value of ≤0.05 was considered statistically significant. Receiver operating characteristic (ROC) curve analysis was performed to determine the sensitivity and specificity of the NLR on admission. The measured values of the SIMs of the DME and non-DME groups were compared to determine optimal cutoff values predictive of the pathogenesis of DME.

## RESULTS

The demographic and clinical characteristics of the groups are shown in table 1. There were no statistically significant differences in mean patient age and female/ male ratio among the DME, non-DME, and control groups (58.22 ± 11.35, 61.92 ± 6.82, and 63.54 ± 5.68 years, and 21/19, 20/20, and 19/21, respectively, p=0.24 and 0.89).

**Table 1:** The demographic and clinical characteristics of the groups

	DME (n=40)	Non-DME (n=40)	Control (n=40)	p
Age (year)	58.22 ± 11.35	61.92 ± 6.82	63.54 ± 5.68	0.240
Female/male ratio	21/19	20/20	19/21	0.894
BCVA (logMAR)	0.64 ± 0.18	0.23 ± 0.11	0.05 ± 0.09	<0.001^*^
IOP (mmHg)	17.89 ± 3.42	18.48 ± 4.24	19.43 ± 3.78	0.236
Duration of DM (year)	8.25 ± 4.83	6.58 ± 2.72		<0.001^†^
HbA1c (%)	7.84 ± 0.87	7.21 ± 0.56		0.042^‡^

BCVA= best-corrected visual acuity; DM= diabetes mellitus; DME= diabetic macular edema; HbA1c= glycated hemoglobin; IOP= intraocular pressure.

* DME vs. non-DME, *p*<0.001; DME vs. control, *p*<0.001; and non-DME vs. control, *p*=0.01.

^†^= DME vs. non-DME, *p*<0.001.

^‡^= DME vs. non-DME, *p*=0.042.

**Figure 1:**
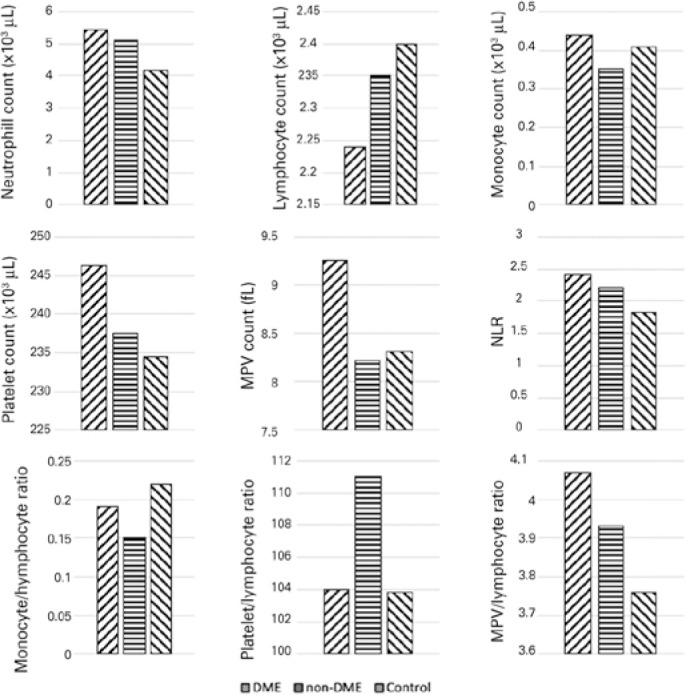
The mean WBC and platelet counts, MPV, and defined ratios.

The WBC and platelet counts, and MPV are shown in figure 1 and summarized in table 2. The mean neutrophil counts of the DME and non- DME groups (5.43 ± 0.70 and 5.10 ± 0.73, respectively) were similar and both were significantly higher than that of the control group (4.17 ± 1.35) (DME vs. non-DME, p=0.31; DME vs. control, p<0.001; and non-DME vs. control, p=0.01). The mean MPV of the DME group was higher than those of the non-DME and control groups (9.26 ± 1.38 vs. 8.22 ± 0.88 and 8.31 ± 0.91, respectively, p=0.01). The MPVs of the non-DME and control groups were similar (DME vs. non-DME, p=0.08; DME vs. control, p=0.02; and non-DME vs. control, p=0.78). The mean lymphocyte, monocyte, and platelet counts were similar among all three groups (all, p>0.017).

**Table 2:** The mean WBC and platelet counts, MPV, and defined ratios among groups

	DME (n=40)	Non-DME (n=40)	Control (n=40)	p
Neutrophil count (×10^3^mL)	5.43 ± 0.70	5.10 ± 0.73	4.17 ± 1.35	0.001^*^
Lymphocyte count (×10^3^mL)	2.24 ± 0.35	2.35 ± 0.42	2.40 ± 0.59	0.734
Monocyte count (×10^3^mL)	0.44 ± 0.20	0.35 ± 0.06	0.41 ± 0.98	0.231
Platelet count (×10^3^mL)	246.31 ± 66.57	237.48 ± 70.20	234.43 ± 50.19	0.892
MPV (fL)	9.26 ± 1.38	8.22 ± 0.88	8.31 ± 0.91	0.010†
NLR	2.42 ± 0.28	2.20 ± 0.39	1.82 ± 0.63	<0.001‡
Monocyte/Lymphocyte ratio	0.19 ± 0.10	0.15 ± 0.47	0.22 ± 0.40	0.292
Platelet/Lymphocyte ratio	103.99 ± 31.26	111.01 ± 33.76	103.80 ± 29.42	0.544
MPV/Lymphocyte ratio	4.07 ± 0.71	3.93 ± 1.21	3.76 ± 1.16	0.178

WBC, white blood cell; MPV, mean platelet volume; DME, diabetic macular edema; NLR, neutrophil/lymphocyte ratio.

* DME vs. non-DME, *p*=0.31; DME vs. control, *p*<0.001; and non-DME vs. control, *p*=0.01.

† DME vs. non-DME, *p*=0.08; DME vs. control, *p*=0.02; and non-DME vs. control, *p*=0.78.

‡ DME vs. non-DME, *p*=0.03; DME vs. control, *p*<0.001; and non-DME vs. control, *p*=0.04.

The mean NLR of the DME and non-DME groups was significantly higher than that of the control group (2.42 ± 0.28 and 2.20 ± 0.39 vs. 1.82 ± 0.63, respectively, p<0.01) and was also significantly higher in the DME group than the non-DME group (DME vs. control, p<0.001; non-DME vs. control, p=0.04; and DME vs. non-DME, p=0.03). The mean monocyte/lymphocyte, platelet/lymphocyte, and MPV/lymphocyte ratios were similar among all three groups (p=0.29, 0.54, and 0.18 respectively). The mean values of the defined ratios are shown in figure 1 and summarized in table 2.

The area under the ROC curve for NLR was 0.680 and an NLR of 2.26 or higher was predictive of the pathogenesis of DME with a sensitivity of 85% and specificity 74%. The results of the area under the curve are summarized in table 3.

**Table 3:** Area under the ROC curve for NLR

Cutoff	2.26
Sensitivity (%)	85
Specificity (%)	74
AUC	0.680
95% CI	0.520 - 0.844
p value	0.035

AUC= area under the curve; CI= confidence interval; NLR= neutrophil/lymphocyte ratio.

## DISCUSSION

There is increasing interest in the association of SIMs with ophthalmological diseases, such as age-related macular degeneration, normal-tension glaucoma, dry eye, central serous chorioretinopathy, and vitreomacular traction^(19,20)^. Although the exact mechanisms underlying the development of these diseases are unknown, inflammatory mechanisms are thought to be responsible. The pathogenesis of DME is clearer than that of other ophthalmological diseases, but there is no report to clarify the usefulness of SIMs. The present study clarified the responses of several SIMs to the pathogenesis of DME and described cutoff values as diagnostic indicators. The results of the present study contribute to the further understanding of DME pathogenesis, diagnosis, and indirectly treatment.

It is known that neutrophils cause progression of in flammation and microangiopathy once adhered to the endothelial cell wall^(21)^. Exacerbation of the inflammatory process causes worsening of DR and the development of DME. Woo et al.^(22)^ reported that a higher neutrophil count is closely associated with DR grade, while the results of this study showed that neutrophil counts were higher in DR patients both with and without DME. The NLR is also a known indicator of systemic low-grade inflammation. Kuang et al.^(23)^ reported elevated NLR in diabetic patients with proliferative DR vs. non-proliferative DR and without DR. Ulu et al.^(24)^ revealed NLR elevation in DR patients and reported a correlation between the NLR and DR grade. In the literature, there are many similar reports of the associations between the NLR and incidence of DR. For example, a meta-analysis of 12 studies with similar methodologies conducted by Liu et al.^(21)^ concluded that NLR is higher in diabetic patients with DR. The most important finding of this study was the demonstration of NLR elevation in DR with DME vs. without DME and healthy subjects. To the best of our knowledge, this is the first report to suggest that the NLR, which is easily obtained through peripheral blood sampling, is a diagnostic indicator of the pathogenies of DME. An optimal cutoff value of the NLR of ^3^2.26 in DR patients was determined in this study. Beyond the diagnostic significance of the NLR, this finding indicates a strong contribution to the pathogenesis that explains DME development.

Another finding indicating a strong contribution to this pathogenesis was the elevated MPV in patients with DME. MPV is a simple, but useful, marker that increases in response to platelet activation^(25)^. With platelet activation, blood clots develop and deliver mediators that promote and sustain local inflammatory responses^(26)^. Buch et al.^(27)^ mentioned that MPV is higher in diabetic subjects with complications as compared to those without and healthy non-diabetic subjects. Citirik et al.^(28)^ stated that diabetic patients have an increased MPV, as compared to healthy subjects, but levels did not change with the DR stage. However, a meta-analysis conducted by Liu et al.^(21)^ concluded that MPV was strongly associated with the severity of DR. The results of the present study showed an MPV increase in DR with DME, which was significantly different from DR without DME and healthy subjects. This result suggests that severe exacerbation in systemic inflammation is needed for the development of DME in patients with DR.

Regarding treatment, intravitreal agents directly or indirectly target the pathogenesis of DME. Intravitreal steroid injections restrict diabetic inflammatory reactions in the microcirculation and stabilize the blood-retinal barrier, while decreasing the production and release of inflammatory mediators^(29)^. Anti-vascular endothelial growth factor agents block the activity of vascular endothelial growth factor, which is released in retinal ischemia, and hypoxia after the inflammatory process^(30)^. In this regard, novel treatment strategies have been developed to break down the inflammatory process. Infliximab is a monoclonal immunoglobulin G1 antibody to tumor necrosis factor alfa (TNF-α) and is used for the treatment of several inflammatory diseases, such as rheumatoid arthritis, ankylosing spondylitis, psoriasis, Crohn’s disea se, and Behçet disease (off-label). Several studies have reported a significant improvement in chronic DME after the intravenous infusion of infliximab^(31)^. In the near future, a greater understanding of the pathogenesis could lead to more widespread use of anti-inflammatory therapies for DME.

The associations between various SIMs and DR have already been revealed. A literature review by Gouliopoulos et al.^(32)^ reported that nearly 20 SIMs (e.g., C-reactive protein, TNF-α and interleukin-6, among others) contribute to the pathogenesis and progression of DR. In comparison, the present study only investigated WBC and platelet counts, MPV, and their ratios as SIMs of the pathogenesis of DME.

There were several limitations to the present study. First, a limited number of inflammatory parameters was studied. Second, the study was limited to only 40 patients in each group. Third, the relevance of WBC parameters for clinical monitoring or individual judgment of the presence of DME could be limited. We would like to emphasize that the relationships among central retinal thickness, WBC parameters, and other SIMs should be supported further prospective studies with larger sample sizes. Nonetheless, the results of the present study are merely initial evidence that this evaluation, which is a relatively simple method, may offer some additional information regarding the risk for the development of retinopathy and macular edema.

In summary, the investigated SIMs were closely associated with the development of DME in DR and easily obtained through peripheral blood sampling. The neutrophil count is higher in DR with and without DME and the NLR is a diagnostic indicator for DME with high sensitivity and specificity. The MPV was significantly higher in DME patients than in those with DR without DME and healthy subjects. To the best of our knowledge, this is the first report of these SIMs of the pathogenesis of DME.
